# Supplementary material for: MetaRibo-Seq measures translation in microbiomes
Source: Nat Commun. 2020 Jun 29;11:3268. doi: 10.1038/s41467-020-17081-z (PMC7324362; doi:10.1038/s41467-020-17081-z)
Supplement: Supplementary file 10 — Supplementary Data 7 [file 41467_2020_17081_MOESM10_ESM.zip › File2/Confidence_High_Taxonomy/14683_out.krona.html]

Javascript must be enabled to view this page.

members
magnitude
magnitudeUnassigned
count
unassigned
taxon
rank

14683\_out

2

2
superkingdom
2

2
phylum
1239

2
class
91061

2
186826
order

1300
family
2

2
genus
1301

species
1305

SRS051244\_contig\_number\_36191
1

1302
species
1

SRS145509\_contig\_number\_21145
